# Spirituality and Moral Injury Among Military Personnel: A Mini-Review

**DOI:** 10.3389/fpsyt.2019.00276

**Published:** 2019-04-29

**Authors:** Suzette Brémault-Phillips, Ashley Pike, Francesca Scarcella, Terry Cherwick

**Affiliations:** ^1^Department of Occupational Therapy, Faculty of Rehabilitation Medicine, University of Alberta, Edmonton, AB, Canada; ^2^Royal Canadian Chaplain Service, Department of National Defence, Edmonton, AB, Canada

**Keywords:** moral injury, military, veterans, spirituality, self, identity, biopsychosocial–spiritual approach

## Abstract

**Introduction:** Moral injury (MI) results when military personnel are exposed to morally injurious events that conflict with their values and beliefs. Given the complexity of MI and its physical, emotional, social, and spiritual impact, a holistic approach is needed. While the biopsychosocial aspects of MI are more commonly addressed, less is known of the spiritual dimension and how to incorporate it into treatment that facilitates restoration of one’s core self and mending of relationships with self, others, and the sacred/Transcendent. The purpose of this study was to gain a greater understanding of the relationship between spirituality/religion (S/R) and MI as experienced by military members and veterans and to consider how S/R might be better integrated into prevention and treatment strategies.

**Methods:** A mini-review of peer-reviewed articles published between January 2000 and April 2018 regarding the relationship between spirituality and MI among military personnel and veterans was conducted.

**Results:** Twenty-five articles were included in the final review. Five themes were identified and explored, including i) Spirituality: A potential cause of and protective factor against MI, ii) Self and identity: Lost and found, iii) Meaning-making: What once was and now is, iv) Spirituality as a facilitator of treatment for MI, and v) Faith communities: Possible sources of fragmentation or healing.

**Discussion:** Findings identified a cyclical relationship between S/R and MI, whereby S/R can both mitigate and exacerbate MI, as well as be affected by it. Seen as a type of S/R struggle, the use of S/R-specific strategies [e.g., forgiveness, review of S/R beliefs, engagement in S/R practices, and (re)connection with S/R communities], integration of S/R perspectives into general interventions, and help from chaplains may support healing, self-regulation, and mending of relationships, moral emotions, and social connection. Further research is yet needed, however, regarding i) S/R orienting systems, interventions, practices, and rituals/ceremonies that might protect against and treat MI; ii) features of individuals who do/do not experience MI; iii) S/R assessment tools and interventions; and iv) ways to maximize the positive contributions of faith communities.

## Introduction

Exposure to morally injurious experiences (MIEs) (1–9) that occur in the course of military service can be mentally and spiritually distressing (1, 10). MIEs, experiences that cannot be justified within a member’s personal and moral beliefs, can leave military members struggling to reconcile seemingly irreconcilable discrepancies between their lived experience, beliefs, values, and worldview(s) (11–14). Occurring in the course of military service, missions, disaster relief efforts, stateside and/or training accidents, drone warfare, or military sexual trauma, exposure to MIEs can be life-altering. Serious psychological problems such as posttraumatic stress disorder (PTSD) and moral injury (MI) can arise (11, 15), leaving military members contending with intrusive thoughts, impulsivity, suicidal ideation, sleep disturbances, or substance use; avoiding experiential triggers; and engaging in maladaptive coping, aggressive, self-harming, self-handicapping, or demoralizing behaviors, all of which can be debilitating (11, 13, 16). More fundamentally, individuals can be impacted at the deepest level of their being (17) and spirit (18). As a result, consideration of the spiritual dimension is needed when trying to understand the impact of exposure to MIEs and prevention and treatment of MI.

### The Human Spirit

The human spirit is “the essential core of the individual, the deepest part of the self” (p. 58) (15). More than characteristics and roles associated with one’s identity (15), the human spirit is a motivating force directed toward realizing higher-order goals and aspirations that grow out of the essential self (19). When exposed to MIEs, a person’s core self, ideals, and perceptions of reality can be shattered and their spirit “broken,” leaving them spiritually and existentially struggling.

### Moral Injury: A Form of Spiritual/Religious Struggle

MI, described as one of several types of S/R struggles (20–24), is associated with questions, disorientation, and tensions about matters of deepest significance that arise within oneself, with other people, and/or with the sacred or Transcendent (15, 25–27). Positively associated with depressive symptoms and negatively with happiness, S/R struggles can impact psychological well-being and health, and cause significant distress (20, 27, 28) and decline (29–31). When a person is unable to resolve S/R struggles, one can experience compromised psychological, social, physical, and spiritual functioning; poor recovery from MI; increased mental health symptoms; and greater risk of mortality. Disconnection from self, others, and the sacred/Transcendent can also occur (22, 26, 31–33). Longer periods of S/R struggle tend to create greater risk (21, 26, 32, 34–38).

### Spiritual/Religious Struggles: Potential for Growth and Resilience

An association between S/R struggles and growth is found in the literature (29–31). Rosner and Powell (2006) reported that there is limited empirical evidence that posttraumatic or adversarial growth occurs due to war, and a paucity of evidence that “adversarial growth” during and after war is specifically due to traumatic events (39). More recently, however, exposure to MIEs has been associated with S/R growth, with some veterans reportedly experiencing renewed faith and more frequent use of prayer as a means of protection (40). S/R commitment, life sanctification, support, and hope have been identified as significant buffers against unhappiness, depressive symptoms, and S/R struggles (28). The most significant growth seems to be related to existential and S/R matters (30, 41, 42).

Growth and resilience related to S/R struggles (including recovery, resistance, and reconfiguration) (43) may be predicated on numerous factors. These include a person’s ability to accept the reality of situations, access supports, draw on S/R resources, make meaning of experiences, (re)affirm a sense of purpose, and (re)engage in positive problem-solving actions (41, 42, 44, 45). Further, a person’s history of S/R struggles seems to be an important factor to consider as those with a lifetime history of S/R struggles appear to have lower levels of well-being. One’s standing on the Big Five and religiousness also seems to be a contributing factor, with higher Neuroticism and Openness, and lower Agreeableness and Conscientiousness, being associated with higher lifetime frequency of S/R struggles and degree of current S/R struggles (27). Identifying those at greater risk of S/R struggles based on their history, personality traits, and religiousness, and facilitating their growth and development, may be beneficial for mitigating the impact of MIEs and development of MI.

This article examines peer-reviewed literature on spirituality as it relates to MI among military personnel and veterans, and its role in the prevention of and recovery from MI. While two scoping reviews have explicitly explored spirituality and MI [Carey et. al.’s review focused on MI, spiritual care, and the role of chaplains (46), and Haight et al.’s review focused on social work research (47)], no review to date has looked more specifically at how MI affects the human person—particularly the spirit (or spiritual) dimension of the self and the importance of using a holistic, biopsychosocial–spiritual model when addressing MI. Spirituality and S/R issues, isolated as key aspects of MI (1), warrant further examination regarding their association with MI.

## Materials and Methods

**Figure 1:**
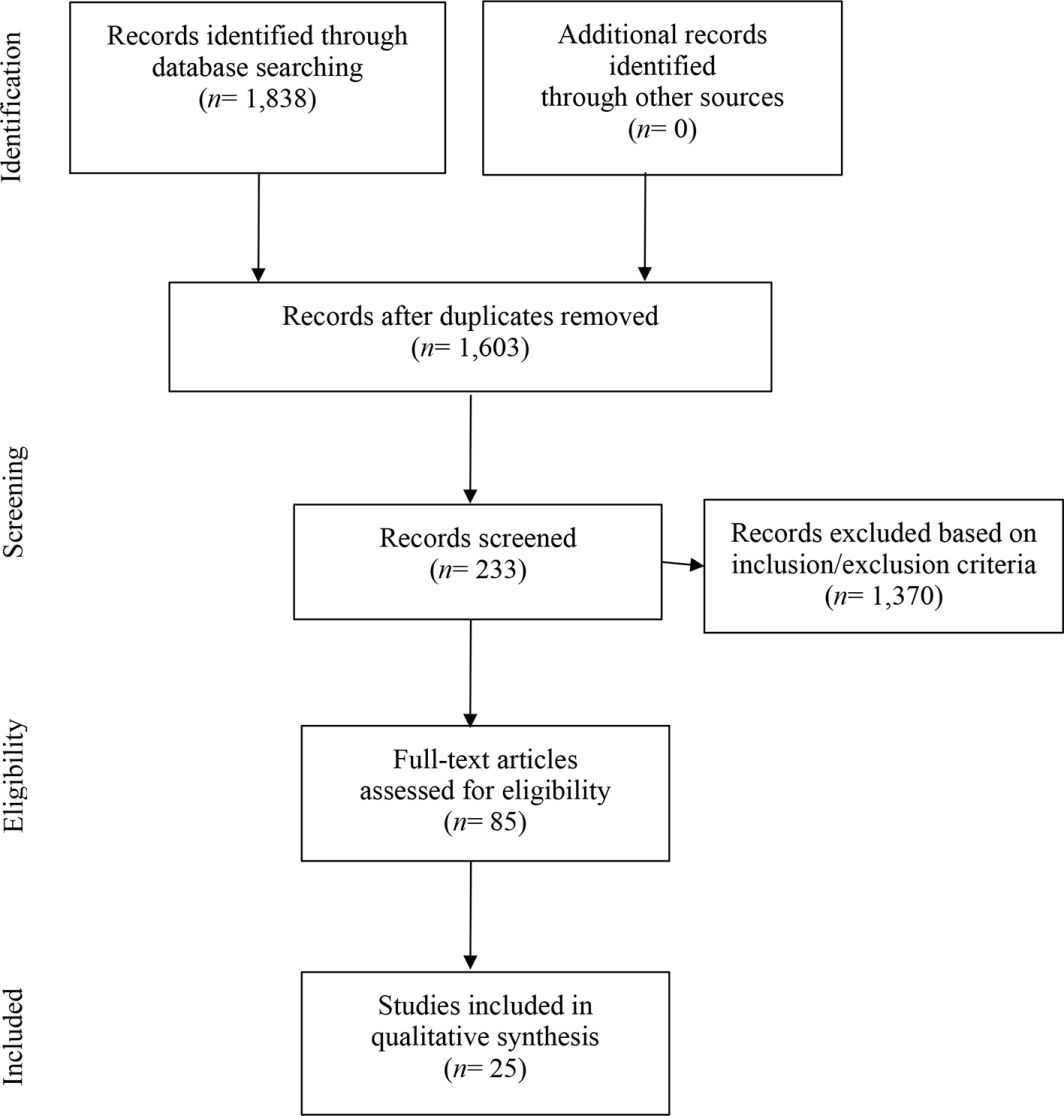
Flowchart of the systematic search. PRISMA flowchart from (82).

The search, selection, and critical assessment of English-language, peer-reviewed manuscripts published between January 2000 and April 2018 were performed independently and blindly by two authors according to the Preferred Reporting Items for Systematic Reviews and Meta-Analysis (PRISMA) guidelines (48). Conducted on January 21, 2017, and repeated on April 22, 2018 to identify new articles published between January 2017 and April 2018, the database searches used SocIndex, MEDLINE (with EBSCO), PsycINFO, Web of Science, and Cumulative Index to Nursing & Allied Health Literature (CINAHL). Keywords included the following: “Moral injur*,” “moral emotions, transgressive acts, morals”; military personnel or naval medicine or military medicine or war or aerospace medicine or soldier* or sailor* or air men or air man or airmen or airman or armed forces or air force or military or naval or coast guard* or submariner* or infantr* or marine corps or marines or army or special forces or warfight* or warfare or land mine* or machine gun* or “United States Department of Veterans Affairs” or Veterans or Veterans Health or army or soldier (49) and spiritual* or faith or theolog* or Muslim or “Bapti* or Buddhis* or religi* or Christian* or Judaism or “belief system*” or meaningful* (50) (**Figure 1**). A manual search of the literature and reference lists was also performed. Data charting utilized data extraction categories suggested by Arksey and O’Malley (51, 52). Concept charting allowed for identification and tracking of overlapping concepts and presenting themes. Discrepancies between authors were resolved through discussion and by consensus.

## Results

**Table 1** presents the studies included in the review, author(s), year of publication, study type, population, and study objectives. The following themes regarding MI and spirituality were identified in the course of the review: i) Spirituality: A potential cause of and protective factor against MI, ii) Self and identity: Lost and found, iii) Meaning-making: What once was and now is, iv) Spirituality as a facilitator of treatment for MI, and v) Faith communities: Possible sources of fragmentation or healing. As for populations represented in the articles, 18 of the 25 articles focused on military populations, 2 considered military members and the role of social work, 3 explored military healthcare/religious professionals, 1 focused on individuals including military personnel experiencing moral stress, and 1 included the development of MI and treatment options as it relates to social work practice in morally complex environments including with military personnel. What follows is a description of the key findings.

**Table 1:** Results of peer-reviewed publications on spirituality and moral injury among military personnel (from January 2000 to April 2018).

Author, year of publication	Study type	Study population	Objective	Spirituality: A Potential Cause of and Protective Factor against MI	Self and Identity: Lost and Found	Meaning-Making: What Once Was and Now Is	Spirituality as a Facilitator of Treatment for Moral Injury	Faith Communities: Possible Sources of Fragmentation or Healing
Blinka et al., 2014 (73)	Qualitative	Military, social workers	Explore role of social work within MI treatment and consider spiritual implications of treating MI		*		*	
Carey et al., 2016 (46)	Qualitative	Military chaplains	To gain an understanding of the role of chaplains within experiences of MI	*			*	
Currier et al., 2015 (67)	Mixed methods	Military	To gain an understanding of how exposure to morally injurious experiences contribute to mental health through meaning making	*		*		*
Doehring, 2015 (54)	Qualitative	Individuals including military members experiencing moral stress	Analysis of moral stress as drawn from the military moral injury literature, considering resilience and the role of spiritual care	*	*		*	*
Drescher et al., 2011 (57)	Qualitative	Health and religious professionals experienced in working with military populations	Construct validation of MI through comparison with semistructured interviews conducted with health and religious professionals	*			*	
Drescher et al., 2011 (57)	Qualitative	Military	Examination of spiritual consequences of MI and PTSD in military veterans		*			*
Evans et al., 2019 (65)	Quantitative	Military	Examined the relationships between potentially morally injurious events, religion/spirituality struggles, and psychological distress	*		*		*
Haight et al., 2016 (47)	Qualitative	Individuals including military members experiencing MI, social workers	Literature review of MI to inform social work research on MI development and treatment options as relates to practice in many morally complex environments including with military populations.	*	*	*		
Hufford et al., 2010 (55)	Qualitative	Military	Exploration of how spiritual fitness can contribute to military unit cohesion, performance, readiness, resilience, and protection	*		*	*	*
Jinkerson, 2016 (53)	Qualitative	Military	Literature review of MI to inform a proposed updated conceptual definition	*		*		
Kopacz et al., 2017 (74)	Qualitative	Military, spiritual care providers	Provide contextual framework for chaplain services provided to veterans, conceptualize the needs of veterans seeking chaplain support, and provide recommendations for providing spiritual care to veterans			*	*	*
Kopacz et al., 2015 (66)	Qualitative	Military, social workers	Inform understanding of role of social work within MI treatment	*		*	*	
Kopacz et al., 2016 (60)	Qualitative	Military	Consideration of complementary therapies as treatment for MI and research strategies to create an evidence base around MI treatments	*				
Kinghorn, 2012 (18)	Qualitative	Military	Consideration of combat trauma and MI from a theological perspective	*				*
Koenig et al., 2017 (68)	Qualitative Case Study	Military	Described a case study employing spiritually integrated cognitive processing therapy	*			*	*
Litz et al., 2009 (11)	Qualitative	Military	Literature review of MI-proposed conceptual framework and interventions	*	*	*	*	*
Malott, 2015 (58)	Mixed methods	Military	Examination of the relationship between morally injurious experiences, religious/spiritual factors, and meaning making in veterans	*		*	*	
Miller, 2016 (61)	Qualitative	Military	Increased understanding of combat veterans’ firsthand accounts of moral, theological, and spiritual struggles following morally injurious experiences	*	*	*		*
Nazarov et al., 2015 (62)	Qualitative	Military	Exploration of association between morality, guilt, and shame	*				*
Pearce et al., 2018 (64)	Qualitative case study	Military	Introduced a new treatment for moral injury, spiritually integrated cognitive processing therapy	*			*	*
Purcell et al., 2016 (40)	Qualitative	Military	Examination of the psychosocial and interpersonal consequences of killing in war and consideration of findings for treatment of military personnel.	*	*		*	*
Rennick, 2013 (59)	Qualitative	Military	Examination of changes in religious and values in Canadian society, role of leaders in moral/ethical experiences and need for enhanced spiritual education for military personnel	*	*	*	*	
Flipse Vargas et al., 2013 (56)	Qualitative	Military	Construct validation of MI through examination of MI themes present in National Vietnam Veteran’s Readjustment Study	*				
Worthington et al., 2012 (63)	Qualitative	Military	Explore mechanisms of development of self-condemnation and how trauma relates to self-condemnation.	*	*		*	*
Yan, 2016 (71)	Mixed methods	Military	To gain an understanding of the impact of spirituality, demographic variables, and MI on physical and mental health of veterans	*	*			
**Total (N = 25)**				**22**	**10**	**11**	**14**	**14**

MI, moral injury; PTSD, posttraumatic stress disorder.

### Moral Injury and Spirituality: Descriptive Summary of the Studies

#### Spirituality: A Potential Cause of and Protective Factor Against Moral Injury

The literature reflects a close association between MI and spirituality (11, 18, 46, 53–65), with spirituality being described as underlying MI and MI having an equally salient impact on spirituality (55, 58, 61–68). A person’s spiritual worldview contributes to the development of MI, with rigid religious principles and expectations potentially enhancing guilt and self-condemnation following exposure to an MIE (63, 66). Hufford et al. suggested that those who are religious may experience greater distress and may be at greater risk of MI due to existential questioning of a Divine being and a “shattering [of] deeply held spiritual beliefs” (p. 76) (55). Currier et al. (67) noted that insult to one’s religious framework or spiritual belief system may result in distress (40, 55, 61, 62, 67, 69). Interviews with combat veterans considering experiences of MI identified loss, questioning, and disillusionment of faith/a higher power as postdeployment experiences (61). As MI can significantly damage the way a person’s values, beliefs, and spirituality guide daily behaviors, consideration of spiritual or theological perspectives may enhance current psychological and medical understandings of MI (18, 54).

Spirituality is also identified both as a protective factor against MI (55, 58, 70, 71) and as a means of coping with MIEs. Hufford and colleagues suggest that spiritual resilience might provide protection from MI in wartime experiences (55). Malott’s (58) survey of 140 Iraq and Afghanistan veterans found that veterans who utilized daily spiritual practices had increased religious coping abilities and levels of forgiveness (58). Further, military personnel whose spirituality was more refined were found to more readily incorporate MIEs into their spiritual framework, potentially reducing the risk of MI (58). Finally, studies suggest that drawing on spiritual practices and Chaplain services during deployment can support spiritual beliefs and offer understanding and context to MIEs (55, 59).

#### Self and Identity: Lost and Found

A loss of innocence, self, and soul during and following deployment has been reported by veterans (61). Cognitive dissonance that arises from discrepancies between an individual’s moral belief system, self-concept, actions, and experiences when exposed to MIEs (11, 59, 72) can disrupt a person’s ability to align their personal sense of right and wrong with that of society (11, 40, 47, 54) and create “maladaptive moral intuitions” of oneself (p. 644) (54). As spirituality enables people to make meaning of events, helping military members “cognitive[ly] [reframe] events as implicitly spiritual experiences” (p. 78) (55) may minimize the risk of MI. In the course of recovery from MI, efforts can be made to help members gain greater self-understanding, self-acceptance, and self-worth through self-reflection (69).

#### Meaning-Making: What Once Was and Now Is

Exposure to MIEs can compromise an individual’s sense of identity, self-worth, and orienting systems, leaving one struggling to find meaning (11, 13, 47, 54, 58, 59, 61, 69, 71, 72). Feelings of unease, self-condemnation, and distress can quickly surface (47, 58, 67). Currier and colleagues found that exposure to more traumatic MIEs is negatively correlated with a person’s ability to make meaning of trauma (67), be that meaning made of the MIE, the impact of the MIE on meaning systems, or attributions of the MIE (11, 47, 53, 55, 59–61, 65, 67, 58, 73). From a syndrome perspective of MI (53), perceived loss of life meaning has been identified as a core symptom within the spiritual conflict domain (61). Deriving meaning from an MIE has enabled veterans to alleviate stress, restore meaning, realign previously established moral frameworks, and influence the likelihood of developing MI (53, 55, 59). Consideration of the MIE and the meaning a member attributes to it is essential when seeking resolution for MI (11), something that Kopacz and colleagues (60) suggest pastoral care can help military personnel with.

#### Spirituality as a Facilitator of Treatment for Moral Injury

The literature outlines treatment strategies required for the prevention and resolution of MI (1, 11, 74, 75). Specific spiritual interventions may be key to alleviating symptoms, reestablishing a stable framework of beliefs, values, and moral code (11, 54), and reestablishing relationships with self, others, the world, and the sacred or Transcendent. This may involve education, modified exposure, self-forgiveness, dialogue with a benevolent moral authority, reparation and forgiveness, (re)connection and (re)engagement with an S/R community, and integration throughout life (1, 11, 74, 76). Spirituality, which is identified as a treatment modality for MI (p. 704) (77), may enhance recovery and healing (11, 40, 46, 54, 55, 58–60, 63, 64, 68, 72, 73, 78).

S/R practices can be incorporated throughout the course of military service. During deployment and prior to returning home, S/R practices and rituals/ceremonies aimed at cleansing, purification, healing, and restoration of relationships with self, others, and the gods/Transcendent have been noted to foster cohesion among military members (55) and facilitate healing and transition to postservice activities. S/R principles and practices—specifically confession and forgiveness from a higher power—are noted to support self-forgiveness (63), which has been identified as a critical component of recovery from MI (11, 63). Further, Drescher et al. (29) highlighted numerous specific S/R intervention strategies: i) forgiveness to facilitate repair of relationships; ii) review of S/R beliefs and engagement in S/R practices that temper anger, rage, and a desire for revenge; iii) prayer and meditation to reduce stress; and iv) (re)connections with S/R communities to reduce isolation, establish social supports, encourage a healthy lifestyle, and facilitate recovery. The following S/R practices can also be incorporated into general interventions (17): i) self-regulation (e.g., through prayer, meditation, yoga, mindfulness, and breathing), ii) self-concept (i.e., enhancing self-awareness through journaling, alignment with a benevolent moral authority), iii) concept of the world (e.g., contributing to making the world a better place through acts of community services, e.g., working at a food bank, soup kitchen, or house-building initiatives), iv) moral emotions (e.g., virtuous living, cultivating a grateful attitude, acceptance, joy), and v) social connection.

Integration of S/R perspectives into general strategies used to treat PTSD and MI is also noted in the literature. This includes the use of spiritual dialogue (77), imagination, and spiritually oriented/integrated cognitive processing therapy (68). Specific spiritually integrated mindfulness, theological reflexivity, and compassion training could be employed to promote recovery and strengthen spiritual fitness and resilience (54, 55). Spiritual-strengthening and meaning-making groups are also suggested (58), as are arts and literature groups to explore S/R and moral dimensions of MIEs (66). Several articles highlighted the importance of chaplains, and pastoral and spiritual care services as a resource for addressing MI (46, 58, 59, 79).

#### Faith Communities: Possible Sources of Fragmentation or Healing

S/R communities can be invaluable resources for military personnel and veterans recovering from MI. Faith-based communities can provide a place and space for members to connect; engage; practice patience, kindness, and forgiveness; spiritually integrate; and reconstruct meaning and purpose (11, 13, 18, 54, 61–65, 40, 68, 73). Conversely, some individuals may experience difficulty engaging in S/R communities due to feelings of guilt, shame, and perceived or real judgment. This can regrettably exacerbate MI symptoms (61, 67). Ideally, however, community can surround military members and veterans struggling with MI so that they can find healing and a renewed sense of meaning and growth.

## Discussion

This mini-review, which aimed to explore the relationship between spirituality and MI, identified a cyclical relationship between MI and spirituality such that S/R was found to serve as both a risk factor for and a protective factor against MI; it can also be directly influenced by MIEs. As a result, consideration of S/R factors, in addition to biopsychosocial elements, is essential when trying to better understand, prevent, and treat MI experienced by military members and veterans.

This review emphasizes the importance of utilizing a holistic biopsychosocial–spiritual approach that leverages S/R resources for the benefit of those who experience S/R struggles such as MI (80) (e.g., the Canadian Model of Occupational Performance and Engagement) (81). Biopsychosocial models more widely used in modern healthcare (46) may be better able to meet the existential and spiritual needs of military members by integrating S/R resources, practices, and tools. This is becoming all the more important as military service becomes increasingly complex and members are impacted at the deepest level of their being (17) and spirit (18) by MIEs not only in the course of missions and disaster relief efforts, but also due to stateside and/or training accidents, drone warfare, and military sexual trauma.

There is growing support for the use of spirituality as both a treatment component of MI and way of addressing the varying values, beliefs, and spiritual needs of military personnel and veterans (11, 40, 46, 54, 55, 57, 59, 60, 63, 64, 72, 76, 79). Helping military personnel and veterans maintain a stable meaning system, ascribe spiritual meaning to MIEs, and access opportunities to discuss moral dilemmas may reduce the risk of developing MI (11, 43, 47, 53, 55, 59–61, 76, 79). This may be facilitated through supportive, nonjudgmental groups and faith communities and engagement in S/R practices and rituals prior to, during, and following military service.

While some evidence exists regarding the relationship between spirituality and MI, further research is yet needed regarding the following: i) how specific S/R orienting systems, interventions, practices, and rituals/ceremonies might protect against and treat MI; ii) features of individuals who do/do not experience MI; iii) S/R assessment tools and interventions, and key time points for their administration; and iv) ways to maximize the positive contributions of faith communities. More robust evidence is also needed to enable the confident use of S/R interventions as it applies to MI in relation to the self, identity, meaning making, S/R struggles, growth, and resilience. Finally, while research has been conducted regarding the way in which S/R may be supportive of members and veterans struggling with MI, further research into supports that social/cultural environments may be able to offer is also warranted.

### Limitations

The scope of the review was restricted to a limited number of indexed peer-reviewed studies that focused on the S/R dimension of MI and were found in the five databases searched. While the search strategy reduced the risk of publication bias, some studies and salient work may have been overlooked. The findings are not generalizable to other groups experiencing MI beyond military personnel and veterans. Further, the subjective nature of concepts presented raises the possibility that personal bias informed thematic findings. To help offset this, the review was conducted by two blind reviewers, reflexivity occurred throughout the research process, and concepts were linked to concrete definitions.

## Conclusion

Spirituality underlies many of the experiences of MI, including changes in identity, meaning making, social supports, and MI symptoms. The findings of this mini-review highlight the need to consider a person’s spiritual fitness and health throughout military service and during treatment of MI and examine how spirituality can be fostered to help build resilience and reduce the risk of MI (17). While acknowledging the limited quality of evidence, encouraging military members and veterans to draw on S/R resources and practices may be a salve to psychospiritual distress. Integration of spirituality as a bona fide modality seems timely. Although much of the literature agreed that spirituality is important for the treatment of MI, more research is needed to understand how to effectively incorporate it into treatment to facilitate healing of MI among military members and veterans.

## Author Contributions

FS and SB-P collected the materials and resources needed for this review. SB-P, FS, and AP analyzed the data. FS and SB-P wrote this article, AP and TC provided subject matter expertise and reviewed and revised each draft of the manuscript.

## Funding

This work was supported by Veterans Affairs Canada, contract number OX170116741401P, and the Royal Canadian Chaplain Service, Department of National Defence. The funding sources had no involvement in the planning, conduction, or evaluation of this study.

## Conflict of Interest Statement

The authors declare that the research was conducted in the absence of any commercial or financial relationships that could be construed as a potential conflict of interest.
